# Evolution after the Revolution: How Classical and Online School Chemistry Teaching Has Changed during the COVID-19 Pandemic?

**DOI:** 10.1021/acs.jchemed.3c00906

**Published:** 2024-02-27

**Authors:** Mária Babinčáková, Paweł Bernard

**Affiliations:** † Jagiellonian University, Faculty of Chemistry, Department of Chemical Education, Gronostajowa 2, 30-387 Kraków, Poland; ‡ Pavol Jozef Šafárik University in Košice, Lifelong Learning Centre and Project Support, Šrobárova 2, Košice 041 80, Slovakia

**Keywords:** Elementary/Middle School Science, High School/Introductory Chemistry, Curriculum, Internet/Web-Based Learning, Testing/Assessment, Professional Development

## Abstract

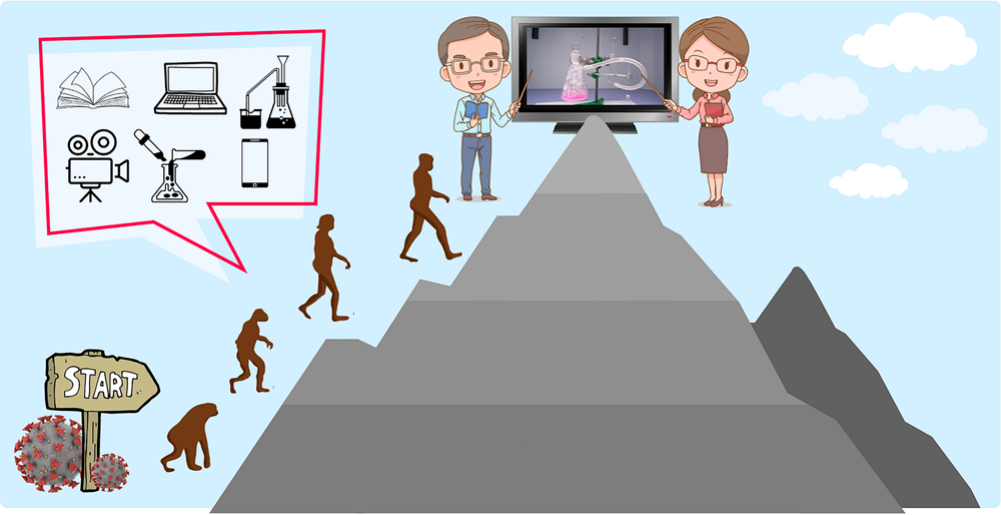

Two years of the COVID-19 pandemic had a tremendous impact on education. In the beginning, teachers were shocked by compulsory online teaching; later, they had to cope with changing restrictions, distance, and blended and hybrid environments. Such a situation was particularly difficult for chemistry teachers, who not only were forced to find a way to organize classes focused on theoretical knowledge but also had to present various phenomena, reactions, and preferably, practice laboratory skills. This paper is focused on changes in chemistry teaching at the secondary school level after two years of the pandemic compared to the first lockouts. The study involved 28 chemistry teachers and their 110 students, all from Slovakian secondary schools, and was based on online questionnaires. Results revealed how online school chemistry teaching changed, what were the teachers’ challenges and attitudes toward online teaching, and how the students perceived online chemistry lessons. It was found that various groups of teachers mastered elements of online teaching at different levels. There are still teachers who struggle with the basics of online teaching but also skilled teachers who can handle many online teaching features. However, all of them still require assistance in their development, covering methodological, technical, and equipment areas. Therefore, results of this study suggest which aspects of online education instructors should pay attention to during pre- and in-service teachers’ training, so skills gained by teachers during the pandemic will not be lost, and which areas of online teaching are beneficial or difficult for students.

## Introduction

**Figure 1:**
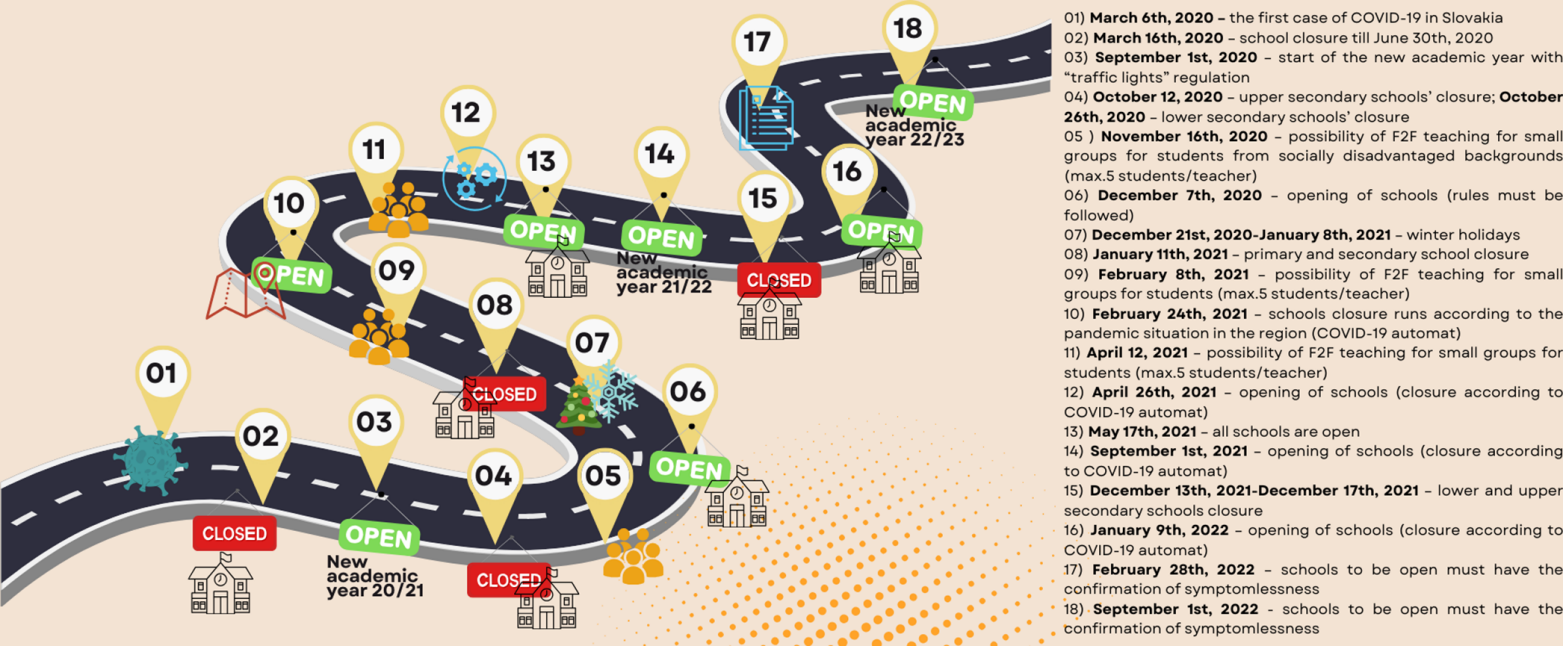
COVID-19 regulation timeline in Slovak schools.

In spring 2020, human lives across the world changed. The COVID-19 pandemic struck and surprised everyone, affecting all daily activities. The natural response was an immediate lockout and later balancing between normal functioning and maintaining the social distance using various partial, local, or regional restrictions. This situation affected the educational process at all levels of education extensively. The first COVID-19 infection in Slovakia was confirmed on March 6, 2020,^1^ and one week later, the Minister of Education, Science, Research and Sport of the Slovak Republic announced a school closure from March 16th. Consequently, restrictions were changing in various areas of the country periodically.^2−4^ Further decisions about schools’ closures are presented in Figure 1.^5^ It shows how unstable the conditions the teachers had to deal with were and how flexible they had to be to ensure the continuity of the educational process.

However, organization of the educational process was not the only challenge teachers faced. As later studies revealed,^6^ only a few of them knew distance education pedagogy, had experience in running online classes, or even had access to platforms with tools for online synchronous group communication. Moreover, during the process of forced online teaching, other limiting factors were revealed:

- weak Internet connections, not sufficient for fluent audio and video communication, probably the most urgent problem all over the world;^7−10^
- lack of hardware–large screen devices for all family members for parallel distance working and learning;^7,10,11^
- teachers’ and students’ overload when regular working hours during the pandemic were canceled;^6,7^
- passivity and low involvement of students during online lessons;^6,8^
- unfair assessment in the online setting.^8,10,12,13^

Despite the general problems, STEM and especially chemistry teachers also struggled with the practical aspects of the lessons. It turned out to be quite challenging because of:

- students’ expectations of authentic laboratory experience;^13−16^
- lack of hands-on experiments suitable for the home environment;^10^
- low availability of supplies for home experiments;^14^
- lack of videos presenting experiments in a way suitable for online classes;^11^
- assessment of inquiry-based activities performed remotely.^14,16^

Nonetheless, many teachers made quite an effort to introduce at least some elements of laboratory experiments for their students. This was evident at both the secondary and tertiary levels. Teachers tried to record their own videos with experiments,^11^ tried to use digitally supported open laboratories,^17^ tried to carry out home experiments based on kitchen equipment,^14,18^ or provided (sent) laboratory equipment and basic chemicals to their students.^14^

Besides the teachers’ dilemmas, the most important voice in the educational process is the voice of students. In addition to hardware and Internet limitations, students reported missing classmates and teachers, unfair assessment, insufficient discussion, and unbalanced workloads.^19−21^ In the context of chemistry classes, students were grateful for teacher’s effort, but on the other hand, they would prefer to have lessons and laboratories at school.^14,21^ They also expected the online lessons to be more similar to classical ones. For instance, they missed a blackboard with meaningful information written and explained by teachers. In many comments, students pointed out that the course of the lessons was too fast. Using teachers’ presentations and videos helped to organize online classes but did not leave much time for asking questions, discussion, and work in groups. Therefore, it was not surprising that students often claimed that their teacher can explain everything better in the classroom. Furthermore, as the pandemic continued and schools started to open, hybrid lessons appeared to be a solution for students attending lessons face-to-face with the combination of students under quarantine, attending lessons online.^22^

Slovak chemistry teachers’ and their students’ dilemmas during the first COVID-19 lockout were described, published, and widely discussed in the summer of 2020.^21^ After two years of the pandemic, in its later phase, in the time of random regional lockouts, the research was repeated. Its aim was to check how the chemistry teaching changed, what were the teachers’ attitudes and challenges, and how students perceived such a teaching approach. Based on the first study and issues raised in other publications, the following research questions were formulated:

- How did the two years of the pandemic affect face-to-face (f2f) and distance chemistry teaching practices?
- What are teachers’ challenges and attitudes toward online teaching after two years of the pandemic?
- How do students perceive online chemistry lessons after two years of the pandemic?

## Methodology

The research described in this paper presents the evolution of online teaching/learning over two years of the pandemic and its impact on later onsite lessons. The research was based on two questionnaire forms, one for teachers and one for students. Questions in both questionnaires were adapted from the initial study run at the beginning of the pandemic^21^ and supplemented with questions from other articles concerning similar issues.^23,24^ The questionnaires for teachers and students are presented in the Supporting Information.

In March 2022, online questionnaires were sent to 50 Slovak chemistry teachers (K7–K12) by email. Teachers taking part in the research were participants of the national project “IT Academy—Education for the 21^st^ Century”. The project was focused on the implementation of information and communications technologies (ICT) in teaching of sciences.^25−28^ During the pandemic, it also offered support with online teaching to teachers in Slovakia.^21^ Subsequently, teachers were asked to send the students’ questionnaire to their students. Teachers were asked to sign the questionnaire with their last name (quotations in the Results and Discussion are presented with random nicknames). Students’ answers were anonymous (quotations in the Results and Discussion are presented with the nicknames they made up), but they were asked to provide their chemistry teacher’s name to match their answers with the particular techniques used by the teacher. Both questionnaires were available for 2 months, and students or teachers could complete them anytime. The questions were answered in the Slovak language, and then, the answers were translated into English. A back translation was provided by another researcher to ensure the text quality. Afterward, interviews were transcribed, coded, and analyzed qualitatively and quantitively. Descriptive answers were translated into English. Additionally, in this case, back translation was used to verify the exactness of the translation. The research was carried out to meet ethical guidelines and requirements of Pavol Jozef Šafárik University in Košice. The participants could reconsider their participation in the study at any stage. The data analysis had mixed character with qualitative characteristics of cases and basic statistics for quantitative data.

Answers from 28 teachers (response rate 56%) were collected (a detailed description is available in Table S1). The teachers had from 14 to 39 years of experience (24 on average, median 22). Sixteen teachers were teaching chemistry at the middle school level (K6–K8) and 12, at the high school level (K9–K12). All teachers were female (based on teachers’ names) reflecting Slovak’s general gender structure in the teachers’ profession.^29^ 110 answers from students were received. The maximum number of students’ answers per teacher was 17; the minimum was 0 (no answers). The average age of the students was 16 years (median 16), with 63 high school and 47 middle school students.

## Results and Discussion

### Teachers’ Perspectives

**Figure 2:**
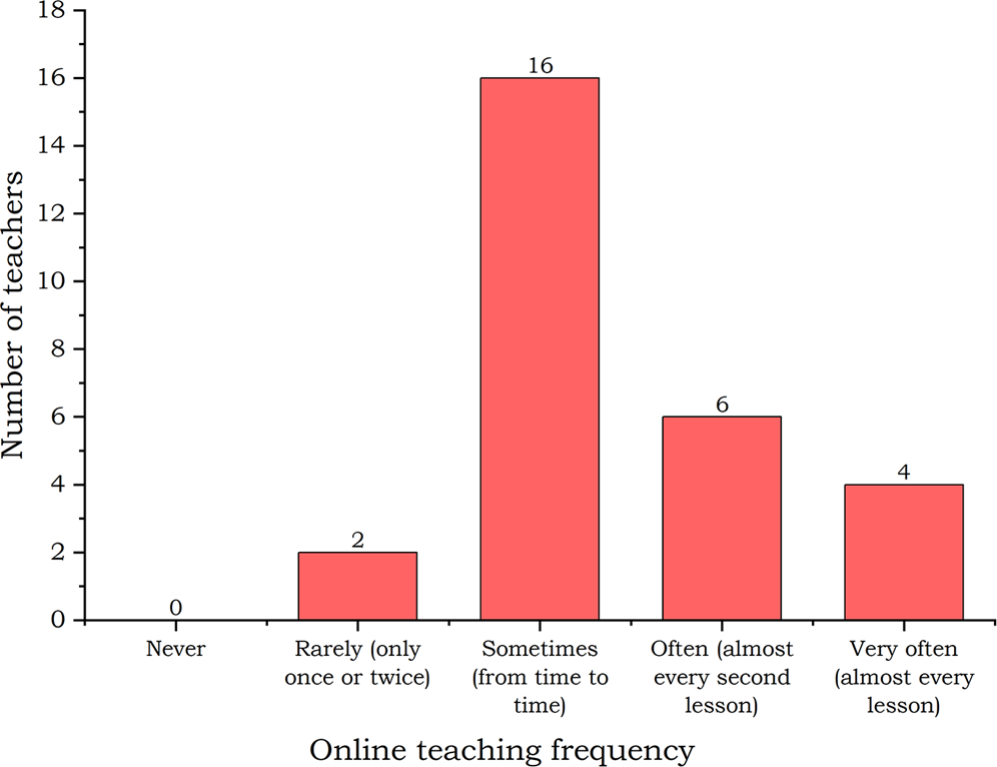
Online teaching frequency: teachers’ answers.

The teachers were asked to estimate how often they were teaching online in the past 6 months (September 2021–February 2022). It is visible (Figure 2) that, after two years of the pandemic, teachers still use online teaching frequently with every teacher incorporating this approach at least once. This reflects the conclusions made during the pandemic when many educators predicted online teaching to become a part of normal education.^30^

#### Changes in Online Lessons

Almost all teachers asked about the most significant change in their classroom practice mentioned an increased use of various information and communication technologies during online lessons. This means broader use of online materials, videos, interactive exercises, or other online tools, such as simulations and virtual experiments. Phoebe mentioned:

*“I gained a lot of new skills and my communication with students improved. We also had online consultation hours for students who were unable to participate in online classes due to health problems or other reasons...Now, I am much more confident.”*

Teachers preparing didactic materials for online lessons in the final stage of the pandemic focused on quality more. As Monika described:

*“The students got used to it* (online lessons—author’s note), *it became more natural—fewer students missed lessons—they had the opportunity to participate in lessons from home—and we increased the quality of our educational videos by reviewing and editing our own materials.”*

What is also very important is that teachers transferred the materials from online classes to their classical lessons and daily practice. Selly wrote: *“I’ve improved in the online environment, used digital teaching tools, and prepared online tests. I still use a lot of these things in traditional f2f teaching.”*

Considering the question “What would you like to change in your online lessons?”, there are three different groups of answers:

- The first group of teachers is focused on the improvement of themselves, i.e., *“I would like to start using a graphic tablet”* (Parvati), *“I want to learn how to make videos presenting theory as well as experiments”* (Ursula), or *“I’d like to learn how to divide students into groups on the online platform”* (Rachel).
- The second group of teachers is focused on the engagement of students. It was well described by Enola: *“Pupils’ activity. The fact that they are hidden behind a computer screen and are often not even present (they are watching movies, chatting with classmates, etc.).”* Gertrude added: *“I would like students to understand that they are learning for themselves...that even if we don’t see them behind that computer when they have their cameras turned off, they will be responsible for their learning.”*
- The third group of teachers is asking for better equipment and better technical support. It means that either they are satisfied with their online teaching or they would like to improve it, but hardware is the limiting factor.

Answers to the question “What is the most acute problem in students’ participation in online classes?” pointed out several main problematic areas: Hardware (11 responses), Internet connection (7 responses), distraction of students (7 responses), students’ laziness (3 responses), and shyness (1 response) (see Figure S1). There is also a group of teachers that had no problems with students’ engagement at all (5 responses). Rachel, on the other hand, highlighted the problem with disabled students who could not fully participate in online classes due to the lack of support for them.

**Figure 3:**
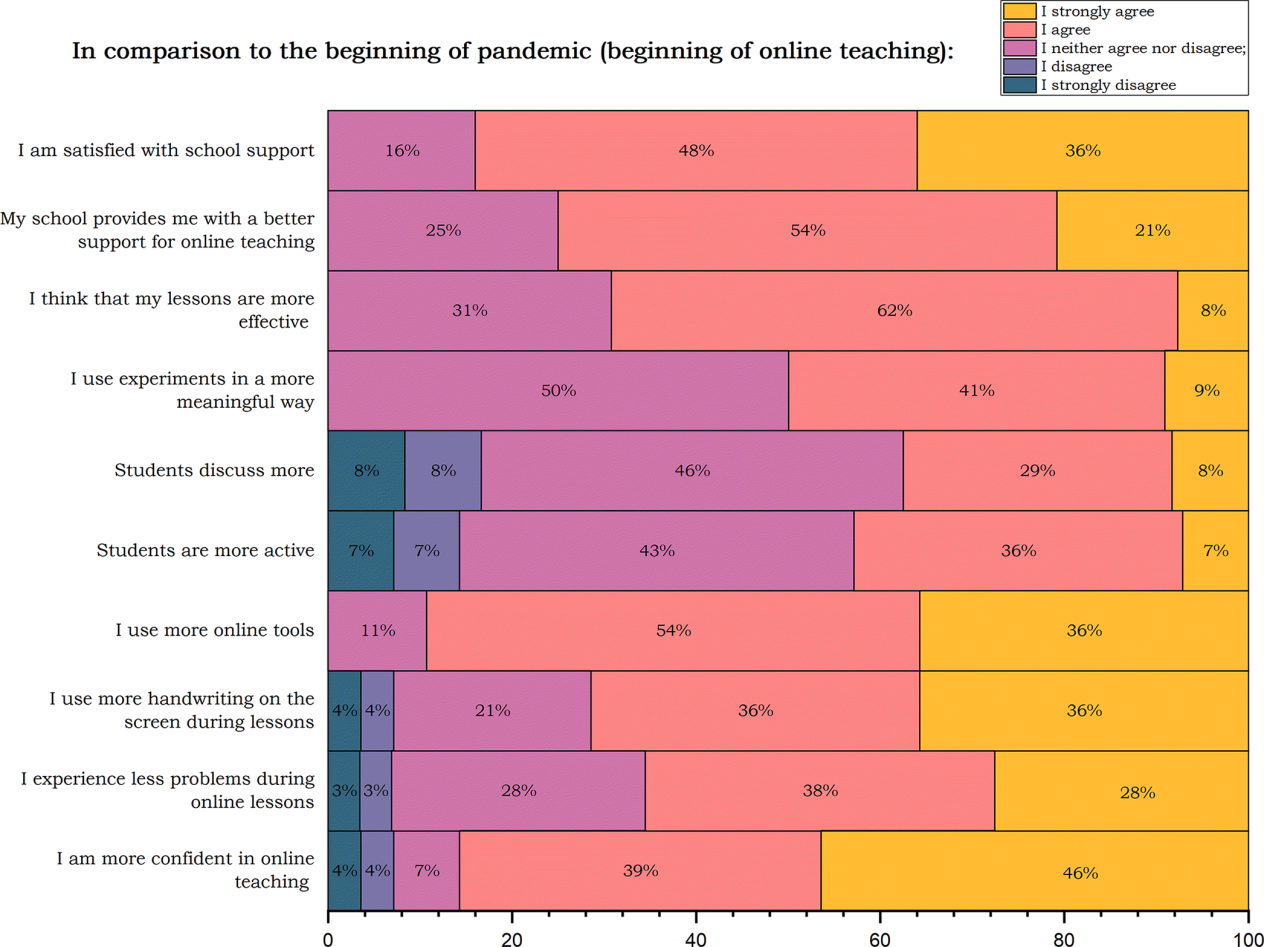
Teachers’ answers and comparison of their current online teaching with the beginning of the pandemic and first lockout.

In the next question, teachers were asked to “Compare your current online teaching with the beginning of the pandemic and first lockout. How do you agree with the sentences below?” (scale: I strongly disagree (1); I disagree; I neither agree nor disagree; I agree; I strongly agree (5)). The sentences and results are presented in Figure 3. Teachers see their improvement after two years of the pandemic. On the other hand, they see room for increasing students’ engagement and active participation during the lessons.

#### Experiments during Online Teaching

**Figure 4:**
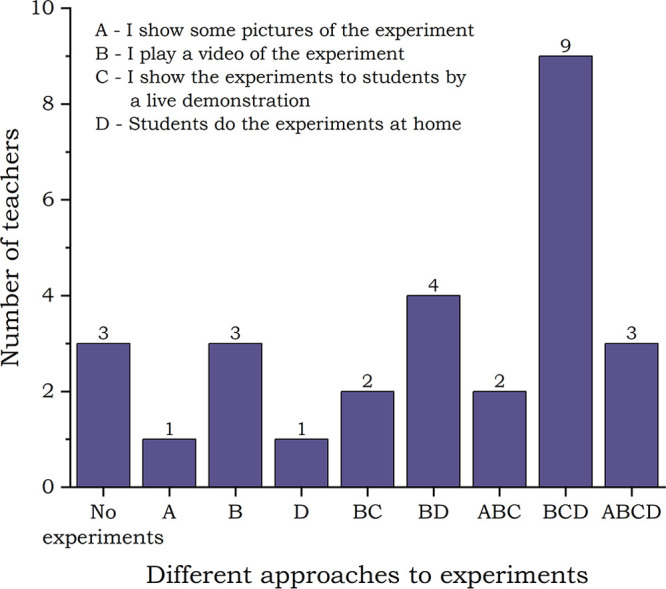
Types of approaches used during online teaching: teachers’ answers.

Teachers were asked if and how they carried out experiments during online classes in the last six months (September 2021–February 2022). They indicated the type of approach used during their online teaching by answering multiple-choice questions. The results are shown in Figure 4.

Almost 80% of the teachers declared changes in their approach to experiments in their online teaching (Question: What has changed in using experiments in your online teaching?). Over time, they created their own databases of videos with experiments and became more skilled at recording their own videos. They also declare a higher frequency of using live experiments during online lessons.

Teachers were asked “What do you consider the biggest change in using experiments in your online teaching?”; Gertrude wrote:

*“Probably the fact that I was more confident to turn on a camera and do the experiment myself, even though it was not perfect. Because at the beginning, when I was teaching from home, I didn’t have the tools or I didn’t have time to prepare them, therefore I played a video via YouTube.”*

Phoebe added: *“Determination to carry out an experiment live and confidence that in the result, it will work as expected.”* Some of the teachers mentioned that they were able to simplify the whole process due to the availability of chemicals or equipment, but there were also negative answers, for example, from Rachel, who experienced a loss of efficiency and energy during the pandemic.

What was the biggest challenge in using experiments in their online teaching? The most frequent answers were as follows:

- recording and using their own videos of experiments (17.8%),
- using experiments more often (10.7%),
- carrying out the experiments by students at home (10.7%).

Parvati summarized what was the biggest challenge for most of the teachers: *“Bring the experiment to the students in a comprehensible and illustrative way so they could discuss it, design and implement themselves at home.”* Any wrote she would like to use more ecologically friendly experiments. Hedwige would like to learn to edit her own videos, and Nataly would like to use a graphical tablet during online chemistry classes.

It was evident that, after two years of the pandemic, the situation in schools has improved. The authorities provided better support for online classes, and the teachers were quite satisfied with it.^31^ Furthermore, the teachers felt more confident in online teaching and, in general, they experienced fewer problems during online lessons. They were able to run their lessons more effectively, present experiments in more complex and interactive form,^21^ and use more online tools and handwriting. On the contrary, students’ participation, activity, and involvement in the discussion still need to be improved.

#### Hybrid Lessons

**Figure 5:**
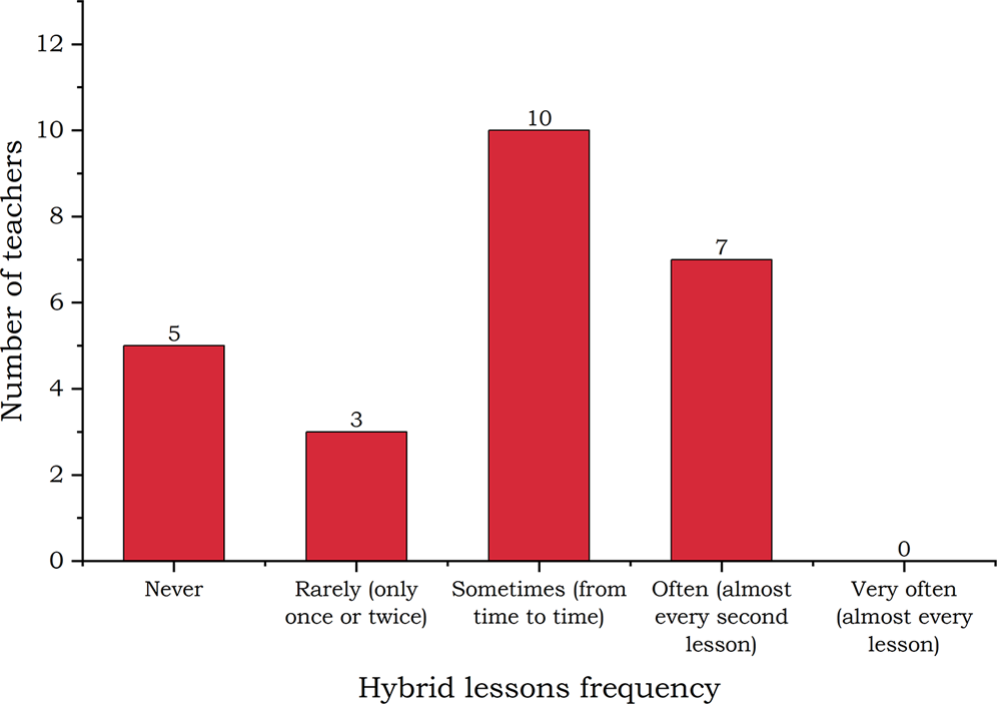
Frequency of hybrid teaching.

Hybrid lessons as a combination of f2f and synchronous distance classes can be a significant step forward in the organization and availability of education. Of course, they require more advanced hardware operated by a skilled and confident teacher. Teachers were asked if and how often they used the hybrid approach with the answers visible in Figure 5.

However, this question was accompanied by an instruction: Could you describe how you organized the hybrid classes? Analysis of those comments pointed out that 3 out of 23 teachers confused hybrid lessons with asynchronous homework, etc. The answers of those teachers were omitted in the analysis. Even though, improvement in this aspect was noticeable. At the beginning of the pandemic, only a few educators were able to implement elements of hybrid teaching, e.g., hybrid protocols.^32−36^ Despite this, due to the obligatory social distancing, many courses were forced to use a hybrid learning model to allow all students to participate in lessons; therefore, the teachers started to implement hybrid teaching in their daily practice.^37^ As a result, many teachers felt confident enough to practice hybrid classes in everyday teaching even after all restrictions were lifted. Moreover, they started to use this approach for consultations and meetings with parents.^38^

#### Staying Online Forever?

Teachers were asked to compare the quality of distance chemical education with full-time conventional lessons, and the results present two points of view. In the first group, teachers see a potential in online lessons—for example, Hermione explained:

*“I think it is possible to teach well in the distance way. If the teacher cares about the education and the results of the students, he will always come up with something useful and interesting. But he must be prepared for a number of unforeseen problems that he will have to deal with (preparation requires a lot of time, technical problems, cheating on the part of students, the need to educate themselves, constantly checking how others do it,... also help others..., exhaustion, frustration, time pressure). And, unfortunately, many times I can only count on myself.”*

In the second group, there are teachers who do not see online teaching as an adequate replacement for f2f teaching. For example, Padma wrote:

*“It* (distance chemical education, note from author) *will not replace the direct education, it can be a supplement, diversification, or partial replacement, but not completely full-fledged, because the student needs to acquire skills in handling, assembling apparatus, assembling models... and perceiving everything with all senses.”*

Rachel added: *“Neither online teaching nor hybrid teaching will replace face-to-face teaching and real experiments.”*

### Students’ Perspectives

#### The Level of Satisfaction with Online Chemistry Learning

**Figure 6:**
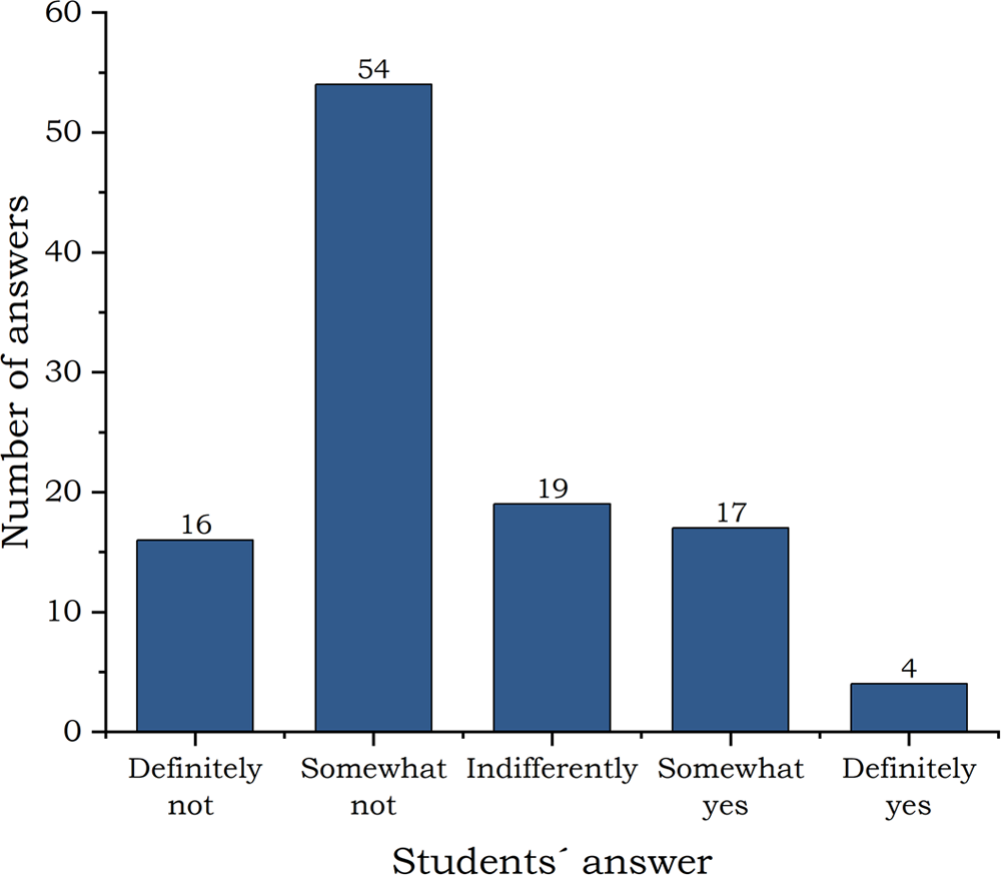
Did you enjoy learning chemistry in the distance way? Students’ answers.

The students were asked if they enjoyed learning chemistry in a distance way and more than half of them (64%) answered that they did not enjoy those lessons (see Figure 6).

Damko, who replied “definitely not”, justified their position as follows: *“Of course, chemistry also includes hours spent in the laboratory, which are missing in distance learning.”* Many other students mentioned missing experiments too. Waka with the answer “somewhat not” added: *“It is difficult to present chemistry in an interesting way when we are online we are distracted by many things and our attention often drops significantly.”*

Only 19% of the students enjoyed distance chemistry lessons. Jane (definitely yes) answered: “*I don’t travel much and I can work at my own pace.”* Smiley (definitely yes) wrote:

*“It’s more casual than at school. Taking notes on a computer is faster and more creative for me. When I need some information or am interested in a given topic, I can immediately find it on the internet. Also, when it is recorded, I can repeat it several times.”*

Home comfort and time flexibility were effective for other students too: *“During distance education, I gained a different perspective on studying in general and the possibility of searching for information and immediate access to the ‘endless’ Internet had its advantages.”* wrote Jakub (somewhat yes).

Next, the students were asked what they liked most about distance chemistry learning (see Figure S3). More than 30% of the students wrote that they do not like anything or they do not like distance chemistry learning. In general, they justified that they more enjoy f2f learning. Those who saw some positives of distance education mentioned, i.e., interactivity during distance chemistry lessons. For example, Viki wrote: *“Interactivity—I like working in online groups with classmates most because we can talk and thus find a common answer to the problem we are solving. I also like videos prepared by the teacher.”* Tamara added: *“Using technology and educational digital websites for learning.”* In general, the students enjoyed home comfort and freedom during online lessons even though they did not enjoy chemistry lessons that much. Domi wrote:

*“Well, this is a difficult question...It is difficult to say that I did not enjoy online chemistry, but it was great to just sit at home in the warmth of the couch and listen to the teacher telling us information about a new topic/substance and just writing notes.”*

The students were able to stay in bed, eat, drink, or do what they wanted. The way of testing also increased the popularity of online education. As Matko mentioned: *“Well, the fact that there are fewer tests and we are writing less in tests.”* Many students emphasized the fact that it was much easier to cheat during online tests. From the other answers, these are highlighted—Smiley: *“The ability to find additional information on a topic which is of immediate interest and ask the teacher questions about it.”*; Janka: *“Silence—Classmates did not interrupt the lesson.”*; Index: *“The possibility to ask questions without everyone looking at me.”*; Natalia: *“Enough time to understand the material, enough time to repeat, and countless didactic materials.”*

From the question “What is the most difficult thing in distance chemistry?” (see Figure S4), it is evident that staying focused during lessons is one of the biggest problems for students. Lenocka answered: *“Stay focused—as my home environment sometimes distracts me. At the same time, due to the online studying, we could not do some experiments practically.”* Also, other students mentioned missing the opportunity to perform experiments. Another common answer was understanding the topic. Domi commented: “*I cannot understand the subject as much as I normally do at school.”* Nelly also commented: In school *“...when we did some more difficult experiments, it was not always easy for me to understand it.”* Another interesting thing students mentioned was the problem with constructing mental models and individual visualization/imagination of processes. Pomaranc wrote what was the most difficult for him: *“Just imagining something that would be shown to me in the laboratory practically.”* Laura also added: *“Imagining certain chemical reactions.”*

**Figure 7:**
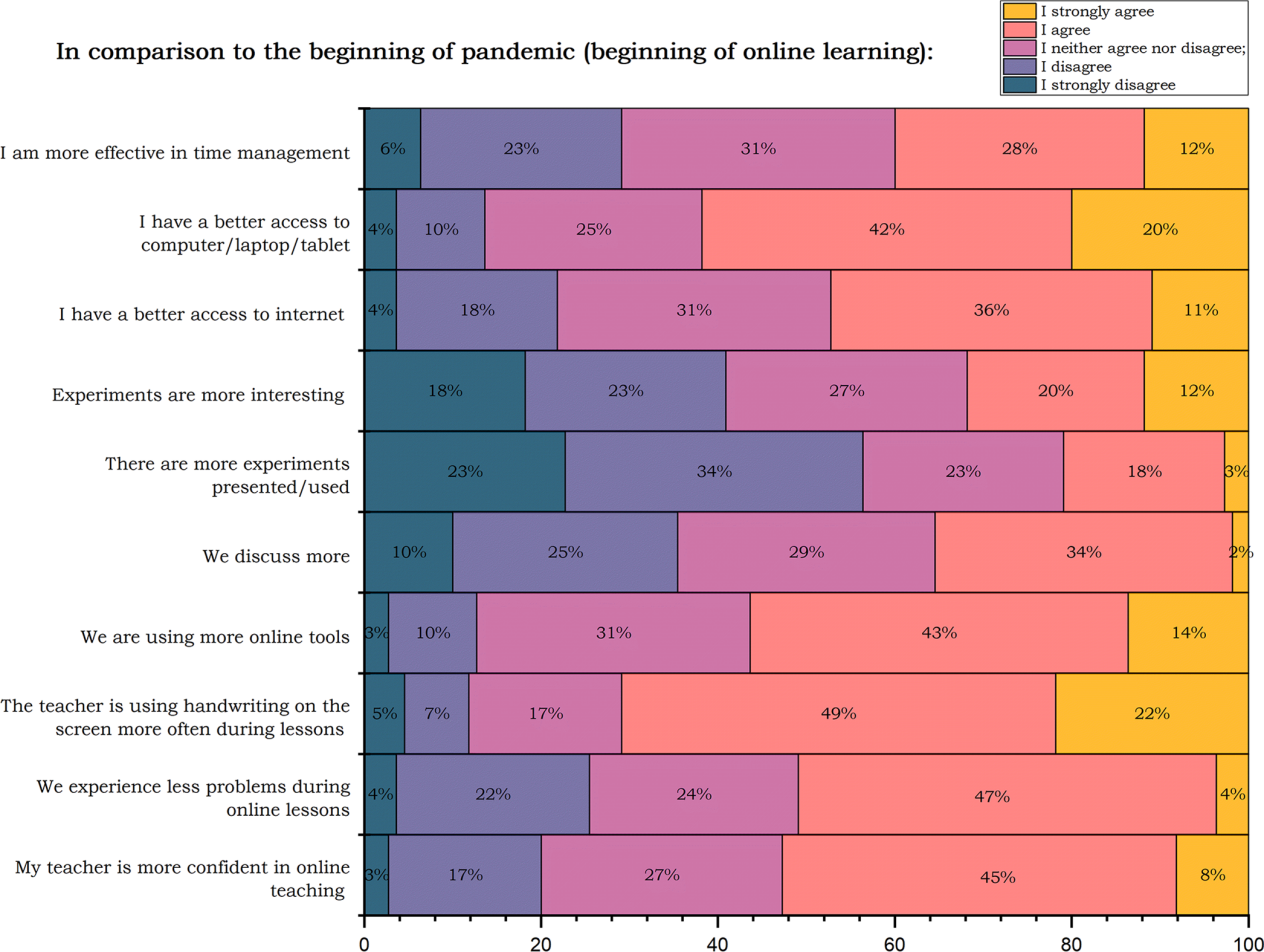
Students’ answers and comparison of their current online learning with the beginning of the pandemic and first lockout.

In the next question, students were supposed to compare their current online learning (March 2022) with the beginning of the pandemic. Their task was to assess if they agree with the presented sentences (I strongly disagree (1); I disagree; I neither agree nor disagree; I agree; I strongly agree (5)). Results are gathered in Figure 7. Students’ answers suggest rather neutral feedback after two years of online learning. The two questions with the most strongly agree/agree answers are connected with the equipment (better access to computer/laptop/tablet) and about the teacher using handwriting.

#### Do Teachers and Students See Things Differently?

**Figure 8:**
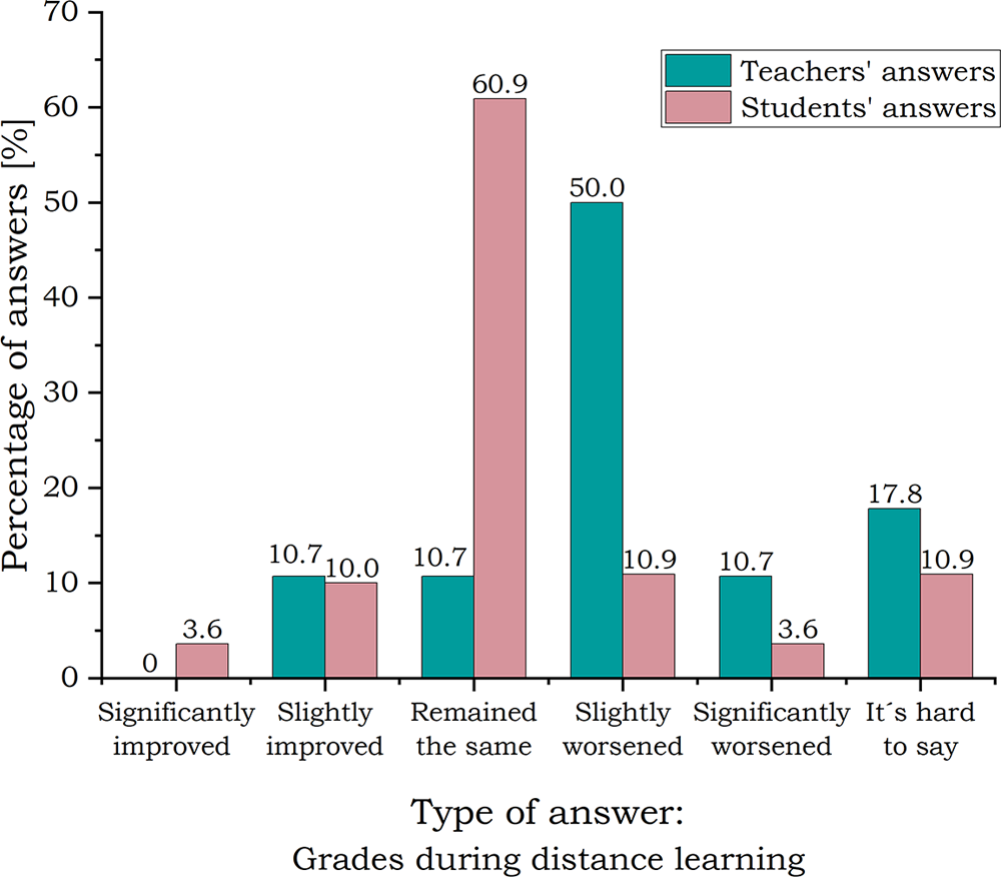
Students’ and teachers’ perceptions of grade changes during distance learning.

The assessment process seems to be the most problematic part of online lessons.^39^ Both teachers and students were asked “How did distance learning affect the grades of your students?” (question for teachers) or “Did distance learning influence your grades in chemistry?” (question for students) (scale for both questions: 1—significantly improved, 5—significantly worsened, 6—hard to say). Students and teachers perceived assessment and grades differently (see Figure 8). More than half of the students, 61% of them (see Figure S5), thought that during the pandemic grades remained the same, while 50% of teachers answered that grades slightly worsened (see Figure S2).

From teachers’ perspectives, perhaps the summative grades remained the same, but teachers were aware that the level of skills and knowledge for those grades decreased. In fact, a reduction of educational requirements caused by the pandemic and forced online teaching was suggested or even forced by authorities in many countries, including Slovakia^40^ and Poland.^41^

As one of the biggest changes in online assessment, the teachers mentioned that, during the pandemic, they tried to evaluate everything students did. They evaluated homework, methods of calculations, presentations, or carrying out experiments more often. Therefore, the teachers also tried to evaluate the process and not only the result. Students saw that too, and for example, Julka (Grades remained the same) wrote: *“It was amazing that the teacher gave us marks for the activity. It helped the overall average a lot. The test marking remained the same which is fine with me personally.”*

On the other hand, the teachers appreciated immediate results from tests run on e-learning platforms. However, they saw that students who were not interested in the subject (especially older ones) dropped out of their lessons completely.

More than 60% of the teachers consider the objectiveness of the assessment to be the biggest challenge. In the same question, the teachers commented that it is difficult to prevent students from cheating, using various resources during tests, and communicating with each other online. Similarly, when students were asked to comment on their answers about the assessment, here, more than 20% of students mentioned that either the assessment was inadequate or unfair or the system enabled cheating (although in the previous question, they answered with “Grades remained the same”). Stela (Grades slightly improved) explained: *“Despite the fact that the professors try to be fair, it is not always fair, because it is not possible to check whether every student is cheating, it is mostly the student’s conscience.”* Mudrlantka (Grades remained the same) wrote: *“It is difficult to prove if we know it or we just read it and wrote it from somewhere during the test.”*

Other teachers saw a great challenge in the use of formative assessment online, especially peer and self-assessment. Student Damian (Grades remained the same) also pointed out: *“It’s fine, but it’s more difficult for some to learn online, so the assessment may be more difficult for some too.”* Branko (Grades slightly improved) mentioned the usage of some form of formative assessment: *“We had a formative assessment only. I was used to a grade but it seems quite good to me.”*

There are more differences in students’ and teachers’ opinions on the development of distance learning during the pandemic. Their views on the most urgent issues were gathered and compared in Table 1. In general, it is visible that teachers’ view is more positive than students’ ones. In objective areas, e.g., using handwriting on the screen during lessons, answers are quite compatible, but in more subjective ones, like confidence in online teaching, differences are noticeable. It shows that teachers are aware of their growth and seem to be satisfied with it; however, they compare only their own classes. On the other hand, students participate in online lessons of various teachers and can compare them too. Perhaps the improvement felt by chemistry teachers was not that high or evident when compared to that of teachers of other subjects.

**Table 1:**
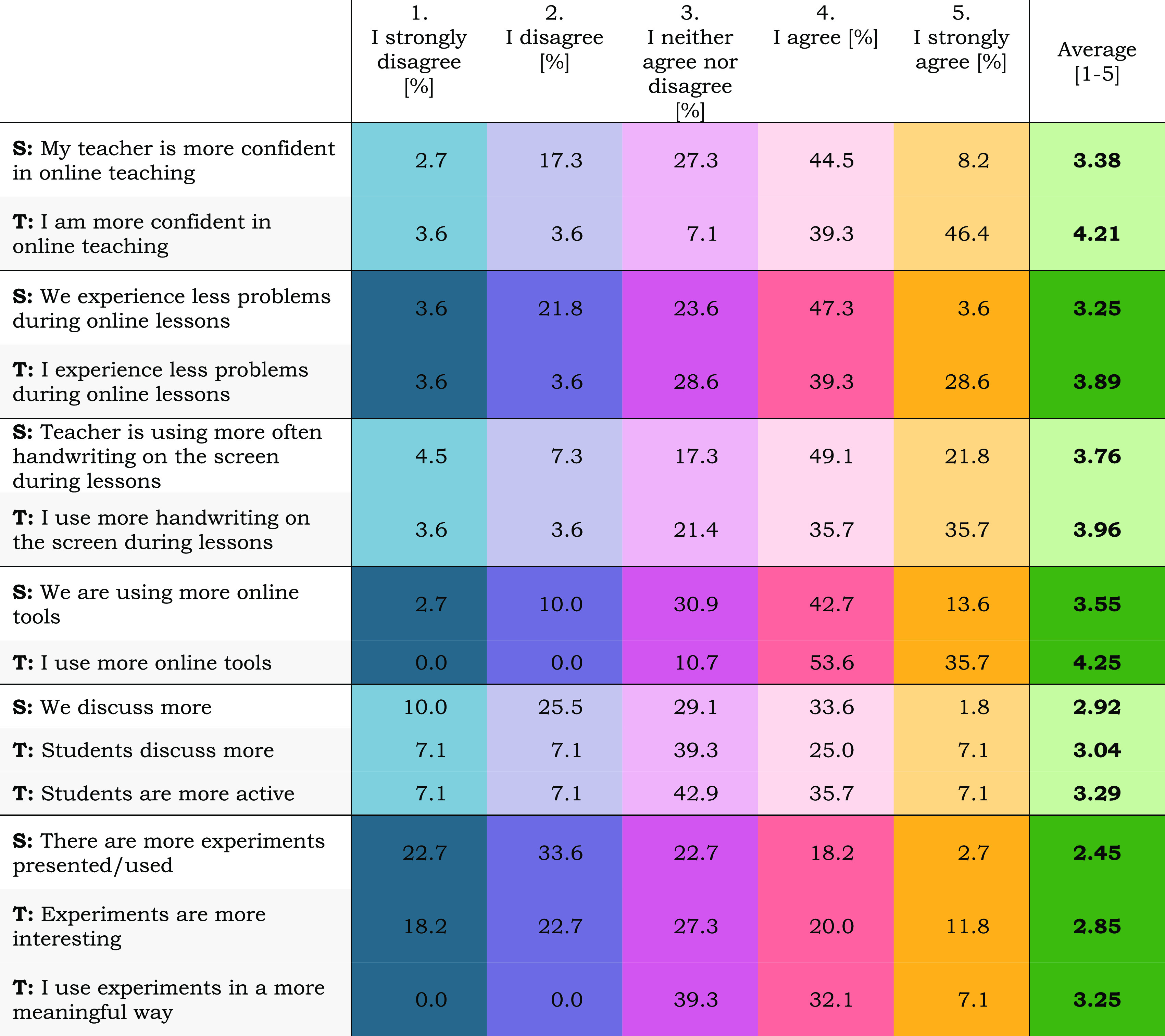
Comparison of Students’ (S) and Teachers’ (T) Answers

## Conclusions and Implications

Even though the pandemic has ended, there is still a lot that chemistry educators can learn from that period. The pandemic happened to be a “game changer” in many areas, especially education. During this period, teachers learned a lot, gained many skills, and started to work with innovations earlier considered uncomfortable.

Almost all teachers reported an increase in the use of ICT, online tools, or experiments during their chemistry classes. Some teachers mentioned that they were able to improve the quality of the online materials, use their own recorded videos, or create a database of learning materials. It may be concluded that there are two different groups of teachers. The first group is still learning the basic principles of online learning and needs help with basic things such as sharing screens, sharing materials, using cameras, or carrying out online demonstrations of chemical experiments. The second group is more advanced, wants to learn new online features such as online peer assessment, working in groups, and doing experiments online simultaneously with students. These two groups have different demands, but they both show the need for further improvement. Areas such as working with conference systems including advanced features, recording and editing of one’s own videos, and effective use of graphical tablets are identified here. Various chemistry related programs and apps should be considered and implemented in teacher training programs.

The students and the teachers wrote that, during the pandemic, the grades did not change or slightly worsened. Additionally, they consider the assessment inadequate and unfair. On the other hand, the teachers used more tools for online assessment which were also more effective in analyzing and providing immediate feedback to the students. Similarly, they assessed not only the students’ knowledge based on tests but also their activity during lessons. The other conclusion for future f2f teaching based on pandemic online teaching practices is using the b-learning approach by providing students online materials which would allow them to keep their own pace or, if they are interested, to study chemistry in more detail. Similarly, the implementation of b-learning at the school level could now be more feasible and provide more students with wider access to the educational process.

There are also drawbacks. One may not forget that learning at a computer and staying focused all the time might be very difficult for students. Moreover, students’ laboratories and experiments should be used in f2f learning and not be replaced by online experiments if it is not absolutely necessary. Students also can develop manual skills during laboratory experiments. Moreover, individual learning may be very difficult for some students.

As mentioned earlier, a positive attitude of teachers eager to learn should be supported by continuous teachers’ training programs or workshops that would respond to the different needs and reflect different levels of the teachers’ skills. Similarly, the school itself–where the whole learning process is happening–plays an unforgettable role in this situation. Equipment and learning platforms should be available to willing teachers in order to create the best environment for them.

Limitations of the study are presented in the SI.

## Supporting Information Available

Teachers’ and students’ questionnaires and all the results; limitations of the study (PDF; DOCX)
