# Supplementary material for: Evolution after the Revolution: How Classical and Online School Chemistry Teaching Has Changed during the COVID-19 Pandemic?
Source: J Chem Educ. 2024 Feb 27;101(3):963–72. doi: 10.1021/acs.jchemed.3c00906 (PMC10939126; doi:10.1021/acs.jchemed.3c00906)
Supplement: Supplementary file 2 — ed3c00906_si_002.docx [file ed3c00906_si_002.docx]

**Supporting Information**

**for**

**Evolution after the revolution: How classical and online school chemistry teaching has changed during the COVID-19 pandemic?**

Mária Babinčáková^a,b^, Paweł Bernard^b^

^a^Jagiellonian University, Faculty of Chemistry, Department of Chemical Education, Gronostajowa Str.2, Kraków, 30-387, Poland

^b^Pavol Jozef Šafárik University in Košice, Lifelong Learning Centre and Project Support, Šrobárova 2, 041 80, Košice, Slovakia

*Corresponding author: pawel.bernard@uj.edu.pl

Supporting Information

Chemistry teaching after 2 years of pandemic - teacher's questionnaire

Chemistry learning after 2 years of pandemic - student's questionnaire

**Results**

*Results from teachers’ questionnaire*

*Results from students’ questionnaire*

[**Figure S1.** The most frequent answers to the question: What is the most acute problem against students’ participation in online classes?](#_Toc155951740)

[**Figure S2** Change in grades during distance learning – teachers’ answers](#_Toc155951741)

[**Figure S3.** Students´ responses to the question: What they liked the most in distance chemistry learning?](#_Toc155951742)

[**Figure S4.** The most difficult things for students during distance chemistry](#_Toc155951743)

[**Figure S5** Students answers to question: Did distance learning influence your grades in chemistry?](#_Toc155951744)

[**Figure S6.** Answers of students to the question: Do you like chemical experiments in distance classes?](#_Toc155951745)

[**Table S1.** Demographic description of teachers participating in the research. Answers for frequency of online classes](#_Toc155951799)

*Limitations of the study*

**Chemistry teaching after 2 years of pandemic - teacher's questionnaire**

Dear colleagues,

thank you very much for your time and for completing this questionnaire. This survey is aimed to find out what has changed in your teaching after 2 years of the pandemic. We hope to find not only your strengths but places to improve as well. The results of this questionnaire will be anonymized. Thank you again.

Questions for teachers:

1. Last name (for merging students' and teachers' answers)
2. Teaching experience (number of years)
3. School level where you teach (multiple-choice)
   - Secondary school
   - High school
4. In the last 6 months, did you run online classes? (1-5 unipolar?)
   - Never (1)
   - Rarely – only once or twice (2)
   - Sometimes – from time to time (3)
   - Often - almost every second lesson (4)
   - Very often - almost every lesson (5)
5. What has changed in your online lessons since the beginning of the pandemic? (open question)
6. What would you like to change in your online lessons? (open question)
7. What is still the biggest challenge for your online teaching? (open question)
8. What is the most acute problem in students’ participation in online classes? (open question)
9. Compare your current online teaching to the beginning of the pandemic and the first lockout. How do you agree with the sentences below: (all 1-5 bipolar) (I strongly disagree(1); I disagree; I neither agree nor disagree; I agree; I strongly agree(5))

- I am more confident in online teaching
- I experience fewer problems during online lessons
- I use more handwriting on the screen during lessons
- I use more online tools
- Students are more active
- Students discuss more
- I use experiments in a more meaningful way
- I think that my lessons are more effective
- My school provides me with better support for online teaching
- I am satisfied with the school support

1. In the last six months, how often have you been using experiments in online teaching? (1-5 unipolar)
   - Never (1)
   - Rarely – only once or twice (2)
   - Sometimes – from time to time (3)
   - Often - almost every second lesson (4)
   - Very often - almost every lesson (5)
2. In the last six months, in which way have you been using experiments in your online teaching? (multiple-choice) - conditional

- I show some pictures of the experiment;
- I play a video of the experiment;
- I show the experiments to students through a live demonstration;
- Students do the experiments at home;
- Some other way: ________

1. In the last six months, how often have you been using data loggers during online teaching experiments? - conditional
   - 1. I never use data loggers
   - 2. Rarely (I used data loggers once or twice)
   - 3. Sometimes (I used data loggers a few times)
   - 4. Often (I used data loggers almost on every second experiment)
   - 5. Very often (I used data loggers almost at every experiment)
2. What has changed in using experiments in your online teaching? (open question)
3. What do you consider the biggest change in using experiments in your online teaching? (open question)
4. What do you consider the biggest challenge in using experiments in your online teaching? (open question)
5. Have you used hybrid teaching during your onsite lessons? (By hybrid teaching we understand mixing face-to-face classes with part of the group, and other students participating in the classes online). (1-5 unipolar)
   - Never (1)
   - Rarely – only once or twice (2)
   - Sometimes – from time to time (3)
   - Often - almost every second lesson (4)
   - Very often - almost every lesson (5)
6. Could you describe how you organized the hybrid classes? (open question) - conditional
7. How did distance learning affect the grades of your students? (1-5 bipolar, 6^th^ hard to say)
   - Grades significantly improved during distance learning
   - Grades slightly improved during distance learning
   - Grades remained the same during distance learning
   - Grades slightly worsened during distance learning
   - Grades significantly worsened during distance learning
   - It´s hard to say
8. What is the biggest change in students’ assessment during your online teaching? (open question)
9. What is the biggest challenge in students’ assessment during your online teaching? (open question)
10. How would you compare the quality of distance chemical education with full-time conventional education? (open question)
11. Is there anything more about chemical education you would like to share? (open question)

**Chemistry learning after 2 years of pandemic - student's questionnaire**

Dear students,

thank you very much for your time and for completing this questionnaire. This survey is aimed to find out what has changed in your chemistry lessons after 2 years of the pandemic. The results of this questionnaire are anonymous (you may provide us with your nickname) so please be honest with your answers. Thank you again :-)

1. Nickname
2. Last name of your teacher
3. Age
4. School level
   - 1^st^ year of chemistry at secondary school and high school
5. Do you enjoy learning chemistry in the distance way? (1-5 bipolar?)
   - Definitely not (1)
   - Somewhat not (2)
   - Indifferently (3)
   - Somewhat yes (4)
   - Definitely yes (5)
6. Why? - for all (open question)
7. Do your chemistry lessons meet your expectations about chemistry as a subject? (1-5 bipolar?)
   - Definitely not (1)
   - Somewhat not (2)
   - Indifferently (3)
   - Somewhat yes (4)
   - Definitely yes (5)
8. Why? - for all (open question)
9. What do you like the most about distance chemistry learning? (open question)
10. What is the most difficult thing in distance chemistry learning? (open question)
11. What do you miss the most in distance chemistry learning? (open question)
12. Compare current online chemistry lessons to those at the beginning of the pandemic and first lockout. How do you agree with the sentences below: (all 1-5 bipolar) (I strongly disagree; I disagree; I neither agree nor disagree; I agree; I strongly agree)
    - My teacher is more confident in online teaching
    - We experience fewer problems during online lessons
    - I have a better access to the internet
    - I have a better access to computer/laptop/tablet
    - Teacher is more often using handwriting on the screen during lessons
    - We are using more online tools
    - I am more effective in time management
    - We discuss more
    - There are more experiments presented/used
    - Experiments are more interesting
13. Do you like chemical experiments in distance classes? (1-5 bipolar)
    - Definitely not (1)
    - Somewhat not (2)
    - Indifferently (3)
    - Somewhat yes (4)
    - Definitely yes (5)
14. Did distance learning influence you grades in chemistry (1-5 bipolar)?
    - Grades significantly improved during distance learning
    - Grades slightly improved during distance learning
    - Grades remained the same during distance learning
    - Grades slightly worsened during distance learning
    - Grades significantly worsened during distance learning
    - It´s hard to say
15. What do you think about assessment in distance chemistry learning? (open question)
16. How has the pandemic affected your approach to learning? (open question)
17. Is there anything more about chemistry learning you would like to share? (open question)

**Results**

Although they agreed that the teacher used more handwriting on the screen (71%), had better access to a computer (62%), and they used more online tools (57%), only half of the students thought that teacher is more confident in online teaching (53%), that they experienced fewer problems during online lessons (51%), or had better access to the Internet (47%). Compared to the beginning of the pandemic, only 40% of students think they were more effective in time management, 36% of students thought they discussed more, 32% of students thought that the experiments were more interesting, and only 21% of students thought that there were more experiments used in online teaching.

***Results from teachers’ questionnaire***

In the next question, teachers were asked: “What is still the biggest challenge in online teaching?” Here, the most interesting answers are presented:

- *“More time for preparation”* (Monika);
- *“To engage more students”* (Celine);
- *“More online demonstrations, not just videos”* (Parvati);
- *“Use of a graphical tablet”* (Nataly);
- *“Working in groups − I have not done it yet”* (Ursula);
- *“Check the writing of chemical equations live during online lessons”* (Hedwige);
- *“Hybrid lessons”* (Olive).


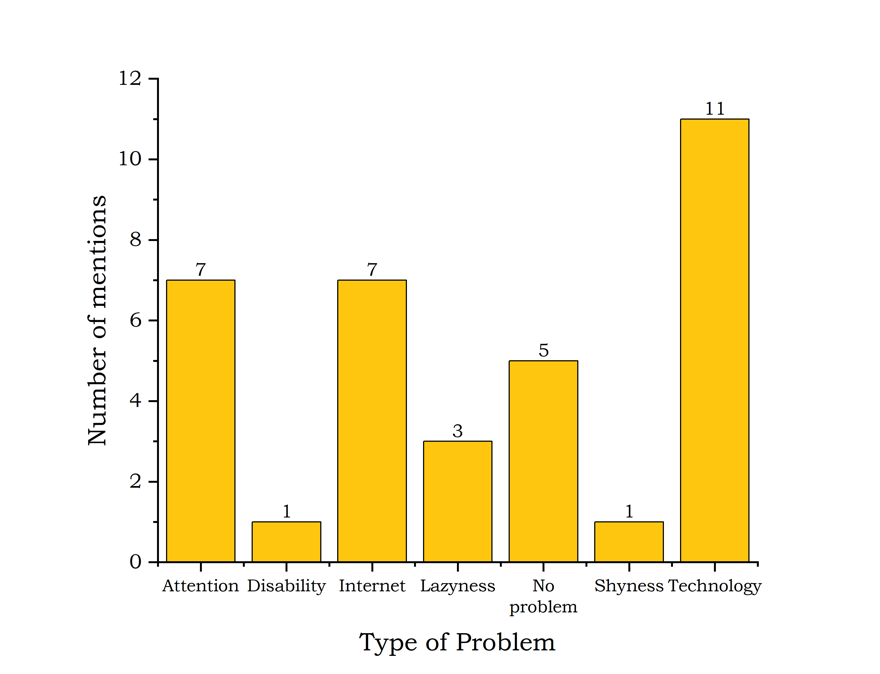


**Figure S1.** The most frequent answers to the question: What is the most acute problem against students’ participation in online classes?


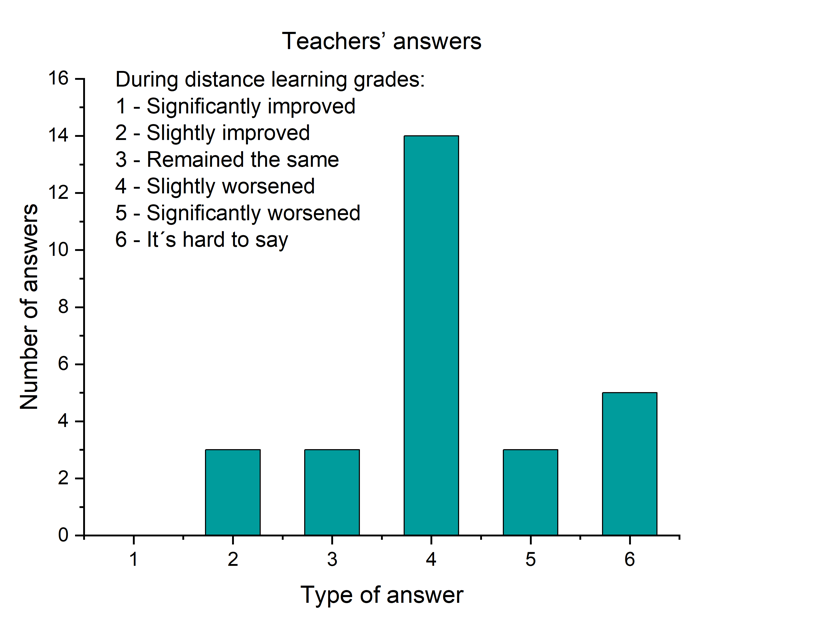


**Figure S2** Change in grades during distance learning – teachers’ answers

***Results from students’ questionnaire***


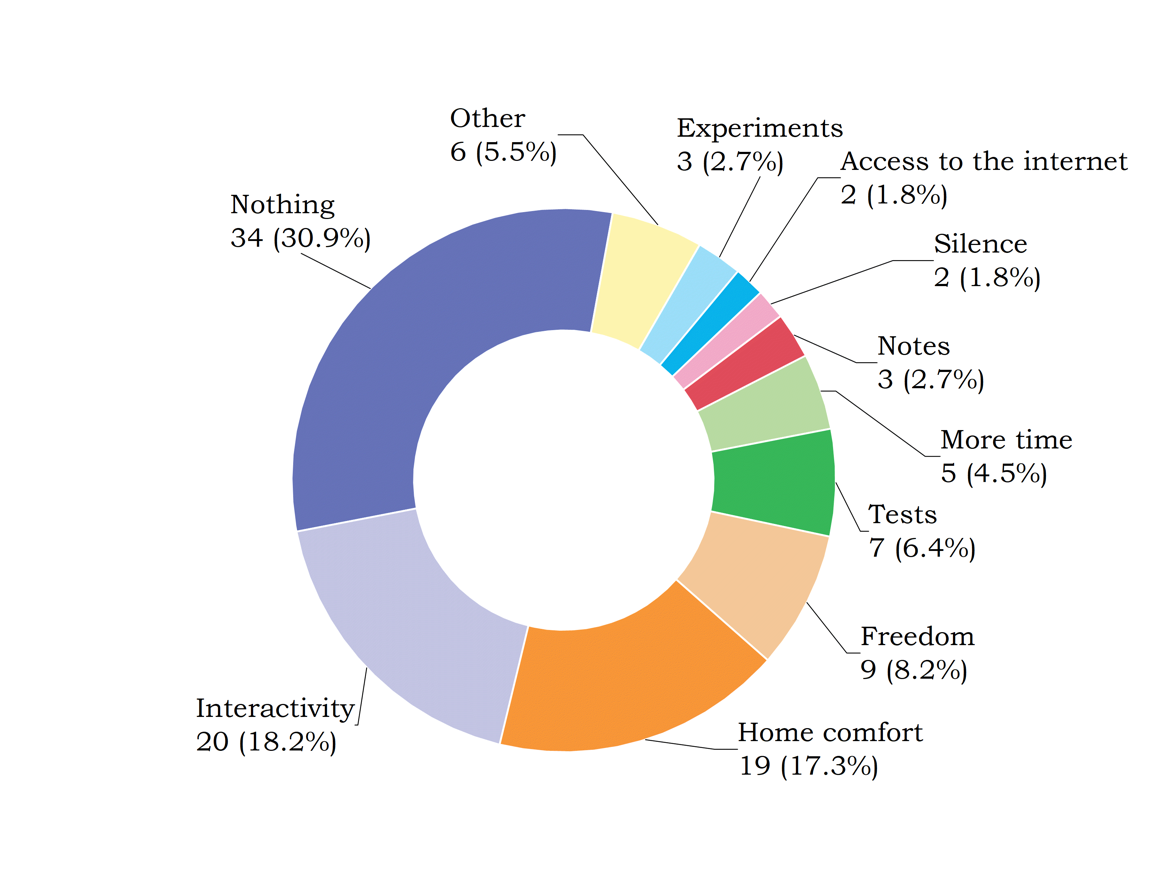


**Figure S3.** Students´ responses to the question: What they liked the most in distance chemistry learning?


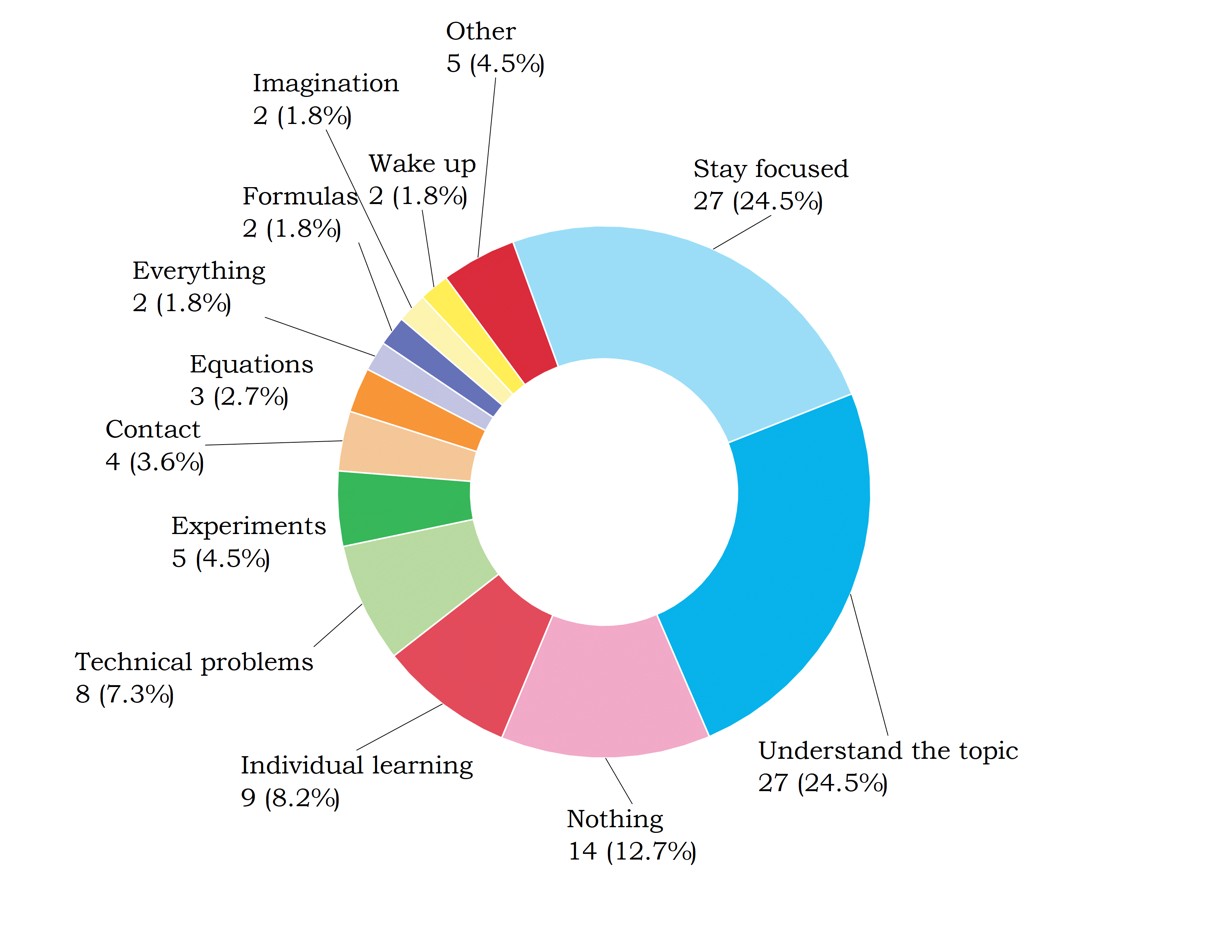


**Figure S4.** The most difficult things for students during distance chemistry


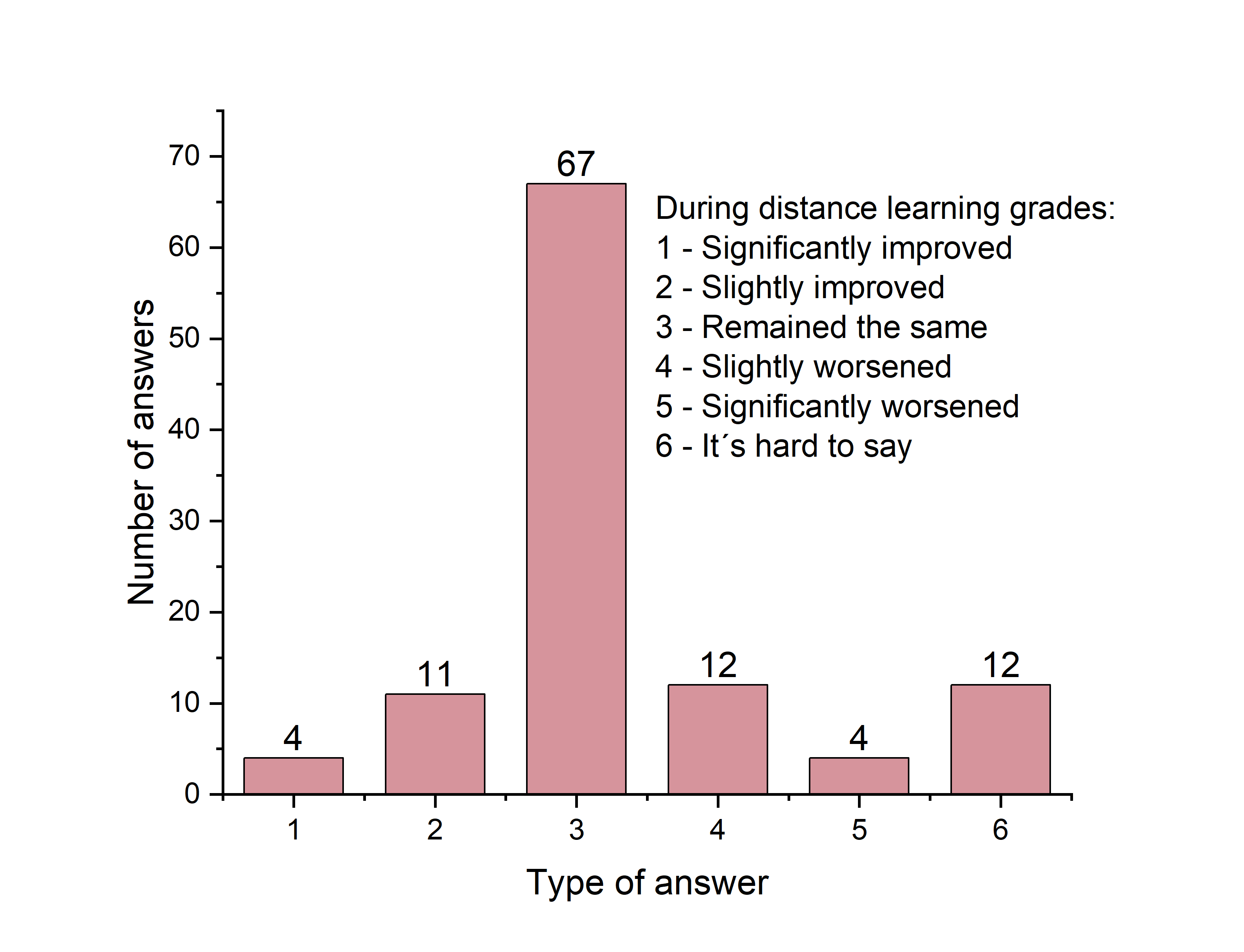


**Figure S5** Students answers to question: Did distance learning influence your grades in chemistry?

Students’ answers to question: Do you like chemical experiments in distance classes? are: Definitely not (12 responses), somewhat not (29 responses), indifferently (37 responses), somewhat yes (20 responses), definitely yes (12 responses) (see Supplementary Figure 11).

Is there anything more about chemistry learning that you would like to share?

- *“Learning chemistry during online classes was, at least for me, the most interesting and effective compared to other subjects.”* (Tamara, 18y)
- *“Some students did not pay attention in class at all and said they have connection problems or something similar. And the teachers didn't believe the students who really had problems.”* (Sima, 13y)
- *“The teacher devoted herself admirably to us. Her lessons were enriched with various interesting and expanding information. The materials she gave us access to were very informative and interesting. All of us who liked the chemistry and biology classes taught by our teacher had the opportunity to consolidate our learning in her classes in order to achieve the best possible results. And she gave us many practical information for life.”* (Natalia, 14y)


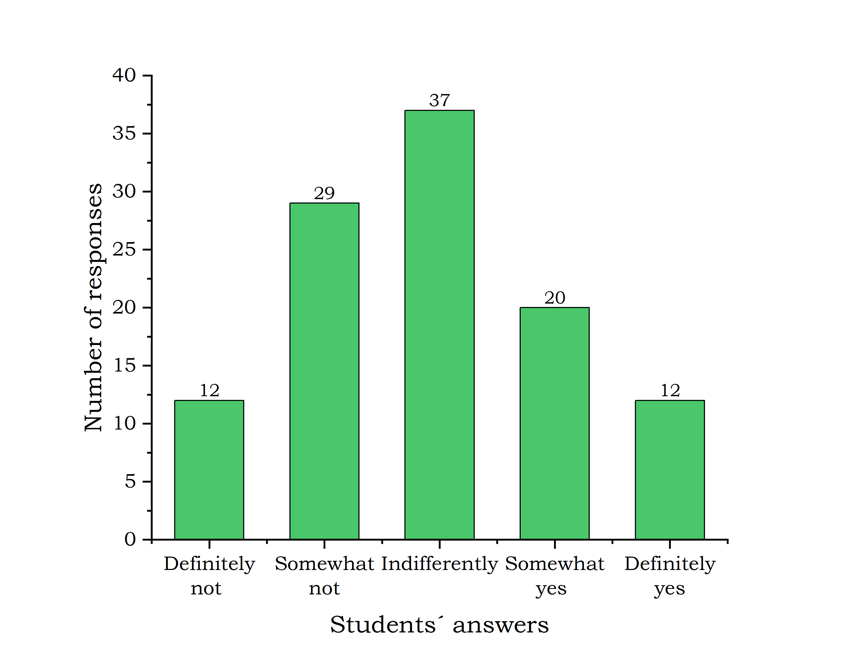


**Figure S6.** Answers of students to the question: Do you like chemical experiments in distance classes? Possible answers: Definitely not (1), somewhat not (2), indifferently (3), somewhat yes (4), definitely yes (5)

**Table S1.** Demographic description of teachers participating in the research. Answers for frequency of online classes (never, rarely – only once or twice, sometimes – from time to time, often - almost every second lesson, very often - almost every lesson).

| **School** | **Teacher**  **Name** | **Number**  **of Students** | **Teaching**  **Experience** | **Frequency of online classes (last 6 months)** |
| --- | --- | --- | --- | --- |
| **Primary School** | Any | 2 | 35 | sometimes |
|  | Barbara | 11 | 25 | sometimes |
|  | Dagmara | 4 | 20 | sometimes |
|  | Fiona | 0 | 30 | often |
|  | Gertrude | 7 | 18 | sometimes |
|  | Ginny | 0 | 31 | sometimes |
|  | Kate | 1 | 22 | very often |
|  | Lucy | 6 | 24 | sometimes |
|  | Maria | 0 | 21 | often |
|  | Monika | 0 | 22 | rarely |
|  | Nataly | 0 | 29 | sometimes |
|  | Noemi | 3 | 22 | sometimes |
|  | Olive | 0 | 26 | often |
|  | Parvati | 7 | 19 | sometimes |
|  | Phoebe | 0 | 39 | often |
|  | Rachel | 9 | 24 | often |
| **High School** | Celine | 17 | 22 | rarely |
|  | Enola | 6 | 18 | very often |
|  | Hedwige | 0 | 28 | sometimes |
|  | Hermione | 0 | 20 | often |
|  | Isabella | 5 | 30 | sometimes |
|  | Jane | 11 | 18 | very often |
|  | Padma | 0 | 14 | very often |
|  | Selly | 2 | 21 | sometimes |
|  | Taylor | 1 | 33 | sometimes |
|  | Ursula | 5 | 22 | sometimes |
|  | Victoria | 3 | 22 | sometimes |
|  | Zannete | 10 | 17 | sometimes |

**Limitations of the study**

The questionnaires were sent to the teachers in March 2022. At this stage, schools had their own responsibility to manage the COVID-19 situation, and regulations for school closures did differ across the country, therefore, participating teachers and students had various online time load when the research was conducted. The questionnaire was made available in an online form which could limit the likelihood of response in case of some teachers and students. Since the questionnaire was prepared in the Slovak, the responses of the participants were coded by a single researcher. The data analyzed in this research are based on answers from 28 teachers and 110 students. Although the group could have been larger, it represents opinions from the whole country and all regions. This study was carried out in Slovakia which may have its specifics and regional aspects. Despite these limitations, we hope that it would guide and help other educators.
